# Blood Transfusion Management for Patients Treated With Anti-CD38 Monoclonal Antibodies

**DOI:** 10.3389/fimmu.2018.02616

**Published:** 2018-11-15

**Authors:** Guido Lancman, Suzanne Arinsburg, Jeffrey Jhang, Hearn Jay Cho, Sundar Jagannath, Deepu Madduri, Samir Parekh, Joshua Richter, Ajai Chari

**Affiliations:** ^1^Tisch Cancer Institute, Icahn School of Medicine at Mount Sinai, New York, NY, United States; ^2^Department of Pathology, Icahn School of Medicine at Mount Sinai, New York, NY, United States

**Keywords:** CD38, monoclonal antibody, daratumumab, isatuximab, transfusion

## Abstract

Daratumumab has proven to be highly efficacious for relapsed and refractory multiple myeloma (MM) and has recently been approved in the frontline setting for MM patients ineligible for transplantation. In the future, expanded indications are possible for daratumumab and other anti-CD38 monoclonal antibodies in development. For several years, it has been recognized that these therapies interfere with blood bank testing by binding to CD38 on red blood cells and causing panagglutination on the Indirect Antiglobulin Test. This can lead to redundant testing and significant delays in patient care. Given the anticipated increase in utilization of anti-CD38 monoclonal antibodies, as well as the transfusion needs of MM patients, it is critical to understand the nature of this interference with blood bank testing and to optimize clinical and laboratory procedures. In this review, we summarize the pathophysiology of this phenomenon, examine the clinical data reported to date, describe currently available methods to resolve this issue, and lastly provide a guide to clinical management of blood transfusions for patients receiving anti-CD38 monoclonal antibodies.

## Introduction

In 2015, daratumumab became the first anti-CD38 monoclonal antibody to be approved by the United States Food and Drug Administration. Its high efficacy and favorable safety profile in recent trials led to expanding indications for relapsed or refractory multiple myeloma (MM) (1–3), and it has recently become the first monoclonal antibody approved in the front-line setting for MM, in transplant-ineligible patients along with bortezomib, melphalan, and prednisone (4).

In addition, there may be further approvals of anti-CD38 monoclonal antibodies in the future. Daratumumab is being tested in various stages of development across a wide variety of cancers as well as in a subcutaneous formulation. Other anti-CD38 monoclonal antibodies are also under investigation including isatuximab, with several ongoing phase 3 trials in MM, as well as MOR202 and TAK079 in early clinical trials for MM (5). A bispecific monoclonal antibody targeting CD38 and CD3, GBR-1342, has also begun a phase 1 trial for MM.

From early on, it was recognized that daratumumab interfered with blood compatibility testing by causing panagglutination in the Indirect Antiglobulin Test (IAT) (6). This was also been shown to be true of isatuximab and MOR-202 surrogates, and indeed is likely to be a class effect rather than specific to any one antibody (7). With increasing numbers of patients receiving anti-CD38 therapy, it is important to recognize this issue in order to prevent delays in obtaining red blood cells and to reduce laboratory costs.

Given the frequency of anemia in patients with myeloma due to marrow replacement by plasma cells, comorbidities (e.g., infections, myelodysplastic syndrome), and various myelosuppressive treatments, blood transfusions are an important part of the supportive care of patients with MM. In this review, we will summarize the pathophysiology of anti-CD38 interference with blood bank testing, review published clinical data, examine various solutions to this problem, and lastly propose a clinical decision algorithm to optimize transfusion management.

## Pathophysiology

CD38 is a transmembrane glycoprotein with various receptor and enzymatic functions (8). It is found in low levels on many cells of both hematopoietic and non-hematopoietic lineages, but has high expression on normal plasma cells. Furthermore, CD38 is highly expressed in nearly all myeloma cells, making it an attractive target for therapy (9). Anti-CD38 antibodies work through a variety of mechanisms, including complement-dependent cytotoxicity, antibody-dependent cell-mediated cytotoxicity, antibody-dependent cellular phagocytosis, induction of apoptosis, and incompletely understood immunomodulatory functions affecting regulatory cells and cytotoxic T cells (8, 10, 11).

**Figure 1:**
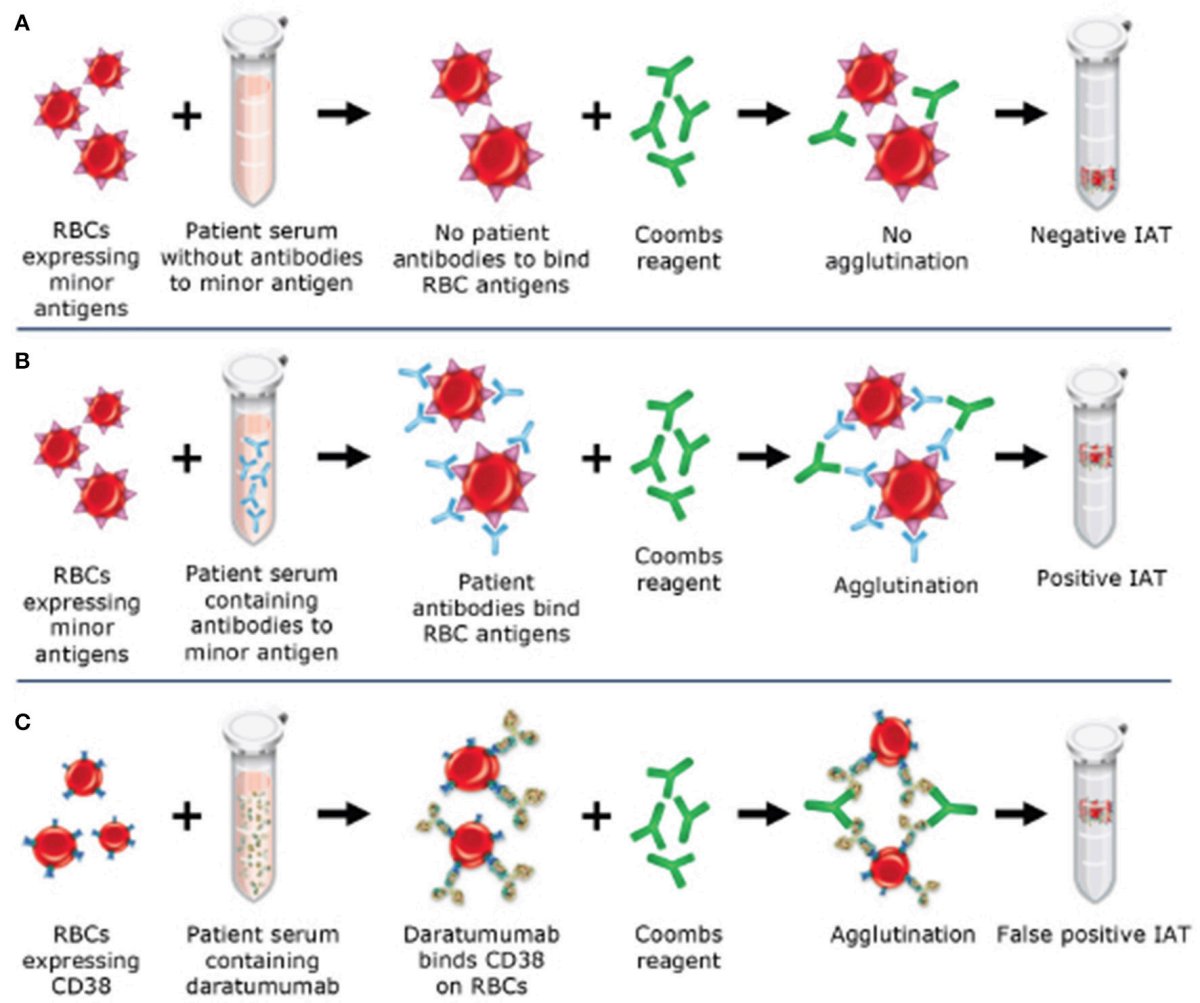
Mechanism of daratumumab interference with the indirect antiglobulin test (IAT). **(A)** Negative IAT in a sample without RBC alloantibodies or daratumumab. **(B)** Positive IAT due to RBC alloantibodies in the sample. **(C)** Positive IAT due to interference from daratumumab, which binds to CD38 on RBCs and causes agglutination with the addition of Coombs reagent.

The indirect antiglobulin test (IAT) utilizes a secondary antibody, antihuman globulin (AHG), directed against the Fc portion of the immunoglobulin molecule to detect antibodies bound to the red blood cell (RBC) membrane. This test is used as part of the RBC antibody screen, RBC antibody identification testing, phenotyping of RBCs for RBC antigens, and in the full crossmatch. CD38 is expressed at low levels on RBCs (12–14), therefore, leading to positive results (agglutination) when plasma from patients on anti-CD38 monoclonal antibodies is used in the IAT. When antibody screening and antibody identification panels are performed as part of pretransfusion testing, anti-CD38 antibody in the patient's serum binds to CD38 on reagent RBCs to cause a weak, usually 1+ panagglutination, in all testing using AHG (gel, tube, solid phase) (see Figure 1) (6). Additional testing is required to identify if RBC alloantibodies are present, leading to delays in the provision of RBCs to the patient for routine transfusions. This cannot be assumed to be a false-positive as many patients have received multiple transfusions in the context of relapsed and refractory MM and may in fact have RBC alloantibodies, necessitating the identification of antigen-negative RBCs for transfusion. Importantly, anti-CD38 antibodies do not affect ABO and Rh(D) typing.

This phenomenon does not routinely occur with a patient's own RBCs, and Direct Antiglobulin Testing (DAT) and autocontrol (AC) on the RBC antibody identification panel are often both negative. In the DAT, the patient's RBCs are reacted with AHG to identify the presence of *in vivo* bound antibodies or complement, while in the AC the patient's own plasma is reacted against their own RBCs to detect both antibodies bound to the red cell membrane and self-reactive antibodies. The lack of detection of antibodies in either test has been shown to be due, at least in part, to downregulation of CD38 on RBCs after exposure to daratumumab (12). Even in the period immediately following infusion of daratumumab, there has been no evidence of any clinically significant hemolysis (15), potentially due to the low expression of CD38 on RBCs.

## Clinical data on impact of Anti-CD38 antibodies on blood typing

To our knowledge, 10 published studies have reported on the results of blood bank testing of patient samples after initiation of anti-CD38 therapy (6, 7, 12, 15–21). In aggregate, these studies provide results for 91 patients (88 treated with daratumumab and 3 treated with isatuximab). All 91 (100%) demonstrated a positive IAT after receiving therapy. In patients who were tested prior to initiating therapy, 5/65 (7.7%) had an RBC alloantibody; the specific antibodies are detailed in Table 1. Six of 43 (14.0%) patients had a positive autocontrol IAT, and 13/67 (19.4%) had positive DAT. One study performed long-term follow-up DAT on three patients who previously had a positive result, and all became negative (17). Three studies reported on time to resolution of positive IAT after cessation of therapy; the durations were 2–6 months (range), median 5 months (range 1–9 months), and median 3.4 months (range 2.1–6.3) (7, 15, 17). Data from these individual studies are presented in Table 1.

**Table 1:** Clinical data on anti-CD38 monoclonal antibody interference with blood bank testing.

**Study**	**No. of patients**	**Anti-CD38 MoAb**	**Pre-existing alloantibodies**	**Positive IAT**	**Positive auto-control IAT**	**Positive DAT**	**Duration IAT positivity**
Bub et al. (16)	5	Dara	n/a	5/5	2/5	2/5	n/a
Carreño-Tarragona et al. (17)	33	30 Dara 3 ISA	anti-D and anti-C (*n* = 2), anti-E and anti-C (*n* = 1)	33/33	n/a	5/21 for Dara 1/2 for ISA	Median 5 months (range 1–9 months)
Chapuy et al. (6)	5	Dara	n/a	5/5	3/5	3/5	n/a
Chari et al. (15)	7	Dara	anti-D and anti-E (*n* = 1), anti-E, K, Jkb, Fya, Fyb S, Knops (*n* = 1)	7/7	1/7	1/7	Median 3.4 months (range 2.1–6.3)
Deneys et al. (18)	14	Dara	None	14/14	n/a	n/a	n/a
Oostendorp et al. (7)	11	Dara	None	11/11	0/11	0/11	Range 2–6 months
Sullivan et al. (12)	13	Dara	n/a	13/13	0/13	0/13	n/a
Subramaniyan et al.(19)	1	Dara	n/a	1/1	0/1	0/1	n/a
Lin et al. (20)	1	Dara	n/a	1/1	0/1	0/1	n/a
Setia et al. (21)	1	Dara	n/a	1/1	n/a	1/1	n/a

*MoAb, monoclonal antibody; IAT, indirect antiglobulin test; DAT, direct antiglobulin test; Dara, Daratumumab; ISA, isatuximab; n/a, not available*.

## Solutions to Anti-CD 38 antibody interference with IAT

Overcoming this interference is possible through a variety of methods, each with its own benefits and downsides. There is no universal solution that can be practically applied in all scenarios, and therefore it is necessary to understand the available options. In this section, we will discuss the methodology, applicability, optimal use, and relative cost of each, as well as the supporting clinical data. A summary is provided in Table 2.

**Table 2:** Approaches for overcoming anti-CD38 monoclonal antibody interference with IAT.

**Method**	**Mechanism**	**Advantages**	**Disadvantages**
DTT	Denatures CD38 antigen on reagent RBCs	Cheap Easy to apply Well-validated and reliable	Denatures Kell antigen; must give K-negative RBCs (unless Kell status known) Destroys other clinically significant minor antigens (Lutheran, YT, JMH, LW, Cromer, Indian, Dombrock, and Knops systems)
Trypsin	Cleaves CD38 antigen on reagent RBCs	Cheap Easy to apply	Denatures several significant antigens (M, N, En^a^TS, Ge2, Ge3, Ge4, Ch/Rg, and Lutheran) Not validated Less reliable than DTT at removing CD38 from reagent RBCs
Papain	Cleaves CD38 antigen on reagent RBCs	Cheap Easy to apply Reliable	Destroys many significant antigens, including MNS and Duffy systems as well as Ch/Rg, Ge2, and Ge4 Due to above, can only be used as a complementary method
RBC phenotype	Antigen profiling of patient RBCs	Only needs to be performed once Provides reliable information for future use Does not require future IAT testing if matched units available	Cannot be done if already started anti-CD38 therapy, or blood transfusion within 3 months Requires extended match to ensure no antibodies or future alloantibody formation Extended-match units may be scarce and better utilized for patients with known alloantibodies
RBC genotype	Antigen profiling of patient RBCs	Only needs to be performed once Provides reliable information for future use Does not require future IAT testing if matched units available Can be performed at any time	Expensive Requires extended match to ensure no antibodies or future alloantibody formation Extended-match units may be scarce and better utilized for patients with known alloantibodies
Anti-idiotype antibody	Neutralizes anti-CD38 antibody prior to IAT	Simple and would allow for normal blood bank testing once anti-CD38 antibody removed Commercially available (for daratumumab)	Expensive Not typically available in blood bank inventory Would require different reagent for each anti-CD38 monoclonal antibody
Soluble CD38 antigen	Neutralizes anti-CD38 antibody prior to IAT	Simple and would allow for normal blood bank testing once anti-CD38 antibody removed Applicable to any anti-CD38 monoclonal antibody Commercially available	Expensive Not typically available in blood bank inventory May be less efficacious than anti-idiotype antibody Would require large amount of soluble CD38 to neutralize therapeutic monoclonal antibodies
F(ab′)_2_ fragments	Fragments preferentially bind CD38 and do not cause IAT positivity	Simple and would allow for routine blood bank testing after application	Not validated Not commercially available
Cord blood/In (Lu) RBCs	Reagent cells lack CD38 antigen	Easy to perform; no additional steps required	In (Lu) RBCs are rare Cord blood cell antigen expression differs from reagent RBCs; therefore, would need to be typed prior to use

### Dithiothreitol and proteolytic enzymes

The most common method of interrupting the binding of anti-CD38 antibody with CD38 receptor is to treat reagent RBCs with dithiothreitol (DTT). The precise laboratory technique has been described in detail elsewhere (22). DTT is a reducing agent that cleaves disulfide bonds present on CD38 receptors. As a result, it can denature CD38 antigen and prevent the antibody from binding. However, this technique also denatures other clinically significant RBC antigens, most notably Kell, but also less immunogenic antigens including those in the Lutheran, Yt, JMH, LW, Cromer, Indian, Dombrock, and Knops systems (23). Therefore, to reduce the risk of a possible hemolytic transfusion reaction due to an unidentified anti-Kell antibody, all patients need to be transfused with Kell-negative blood, unless they are known to be Kell-positive. Clinically significant antibodies in patients with antibodies against the DTT-sensitive antigens such at the Cartwright blood group system (Yt) would be missed using this test method and put the patient at risk of a hemolytic transfusion reaction.

Although DTT treatment is a technically straightforward method to perform in the laboratory, it can require more than 2–4 h to perform manually, requires properly trained medical technologists using standardized procedures, and DTT is noxious and should be used under a hood. The cost of the reagent is minimal, but implementation is usually encumbered by the absence of resources that are not routinely available in many hospital blood banks.

Treating RBCs with DTT is very reliable and has been validated in an international multi-center study (22). In this study, 25 centers each received two blood samples, one spiked with daratumumab and another spiked with daratumumab plus a clinically significant RBC alloantibody (either anti-s, anti-D, or anti-Fy^a^). 24/25 (96%) of centers observed daratumumab interference in the first sample with their routine tests, and all of them could resolve this using DTT-treated reagent RBCs. In the second part of the study, 100% of the study sites could identify the RBC antibody after removal of daratumumab interference with DTT. Most of the centers surveyed at the conclusion of the study found the method to be simple and reported they would use it in the future as their standard of care. However, it is important to note that these were all academic medical centers or blood center reference laboratories, and feasibility for smaller community laboratories may differ.

Treatment of RBCs with the proteolytic enzymes trypsin or papain has not been studied to the same extent as DTT and these methods are unlikely to replace DTT at this time. Chapuy et al. demonstrated that 2% trypsin reduced daratumumab binding to CD38-transduced HL-60 cells by 40%, compared to 92% for 10 mmol/L DTT (6). Trypsin does not degrade Kell antigens, but does destroy a number of other clinically significant antigens including M, N, En^a^TS, and the less immunogenic Ge2, Ge3, Ge4, Ch/Rg, and Lutheran antigens (24). Additional studies are needed to determine the advantages in clinical utility and laboratory operations using trypsin compared to DTT.

Papain was successfully used to eliminate IAT interference from daratumumab and isatuximab in all 33 patients in one study (16). The authors were able to identify Rh group antibodies in all three patients with pre-existing alloantibodies. Hypothetically, papain could be used for quick identification of Kell antibodies as it does not denature Kell antigen, but this was not tested in the study. Papain does degrade antigens from the Duffy and MNS blood group systems, as well as several minor antigens including Ch/Rg, Ge2, and Ge4 (24), so its use would mainly be complementary to other approaches. In the study, papain was used in conjunction with phenotypically matched RBCs for safe provision of transfusions. If used in parallel with DTT, it could overcome the limitation of having to provide Kell-negative blood when using DTT alone. This would, however, increase the complexity of laboratory procedures and may not be practical in a routine setting.

### Typing of RBCs

Extended phenotyping and genotyping of patient RBCs are effective, albeit expensive, methods for safely providing compatible blood. Phenotyping must be done prior to initiation of anti-CD38 antibody therapy and in the absence of both RBC transfusion in the prior 3 months and positive DAT. Extended phenotyping assesses, at a minimum, the most common immunogenic antigens, namely those in the Rh, Duffy, Kidd, Kell, and MNS blood group systems (18). Genotyping for RBC antigens can be performed at any time during therapy and can provide more comprehensive detail than phenotyping, particularly regarding minor antigens (25). Genotyping is expensive and requires at least 1 week of turnaround time in most cases.

Information collected from RBC typing is stored and then used to provide phenotypically matched blood for future transfusions. Providing antigen-matched RBCs can be a challenge for blood banks with smaller inventories and may unnecessarily use scarce resources that are better utilized for patients more likely to have broad alloimmunization (e.g., sickle cell anemia). Use of extensively matched RBCs may not be necessary if DTT treatment is used; a negative antibody screen after DTT treatment would only require RhD and Kell compatible blood.

In practice, transfusion with phenotypically matched RBCs has been very safe. Chari et al. (15) reported on transfusion outcomes in SIRIUS, a phase 2 trial of single agent daratumumab for treatment-refractory MM (26). In this study, 47 patients received a total of 147 units of packed RBCs without any transfusion reactions or evidence of hemolysis (15). In-depth analysis of two clinical sites showed that exclusively using phenotypically-matched RBCs resulted in no adverse transfusion reactions, hemolysis, or development of new alloantibodies.

Deneys et al. reported on 11 patients at their institution who had been enrolled in daratumumab clinical trials and required transfusion (18). These patients were assigned phenotypically-matched RBCs that were cross-match compatible when DTT treated donor RBCs were tested against the patients' serum. Patients received between 2 and 44 units of RBCs and there were only 2 mild transfusion reactions (1 fever, 1 erythema). Another short letter reported five patients in a daratumumab clinical trial who received between 1 and 20 units of RBCs on the basis of extended phenotyping and/or genotyping; again, no adverse events were reported (16).

It should be noted that these data, while encouraging, were obtained from clinical trial contexts in large academic centers. The feasibility of RBC-typing and provision of antigen-matched RBCs in the community setting should be assessed on a case-by-case basis.

### Anti-Idiotype antibody and soluble CD38 receptor

The most direct approach to prevent IAT panreactivity is to neutralize the anti-CD38 antibody in the patient's serum prior to conducting the IAT. This can be done with a reagent antibody directed at the specific anti-CD38 monoclonal antibody or a decoy soluble CD38 receptor. The efficacy of these techniques was demonstrated in two studies in which blood samples were spiked with daratumumab and then treated with anti-daratumumab antibody or soluble CD38 receptor (6, 7). Both were able to eliminate the interference with the IAT, although in one study the soluble CD38 receptor was perhaps slightly less effective (6). Importantly, these did not interfere with known alloantibodies present in the spiked serum.

Recently an anti-daratumumab antibody was approved to similarly mitigate the interference of daratumumab on serum immunofixation tests (27). However, these reagents are expensive and a specific anti-idiotype antibody would have to be designed for each new anti-CD38 monoclonal antibody. For soluble CD38 receptor, large quantities would need to be used to reliably overcome the anti-CD38 antibody concentration in the patient's serum. A recent correspondence reported the use of F(ab′)_2_ fragments, produced by digestion of daratumumab with pepsin, to preferentially bind CD38 on reagent RBCs and prevent daratumumab interference (28). The authors were able to detect known RBC allo-antibodies in several samples using this technique. This method may prove useful but requires further validation and widespread commercial availability. For the foreseeable future, these are likely to be utilized only in a research setting.

### CD38-negative RBCs

RBCs lacking CD38 antigens do not bind anti-CD38 monoclonal antibodies and therefore do not demonstrate panagglutination in the IAT. It has been shown that cord blood may lack CD38 antigen, which was exploited in pre-transfusion testing to successfully screen for antibodies at one clinical site; 17 units of RBCs were transfused without adverse events (29). However, cord blood may have different antigen expression than RBCs routinely used in blood bank testing (e.g., P1, Lewis), and would need to be typed prior to use (30). Similarly, the rare In(Lu) RBCs which are Lu(a– b–) do not react with daratumumab (31). However, these are not readily available and therefore not a practical solution.

## Clinical decision-making

**Figure 2:**
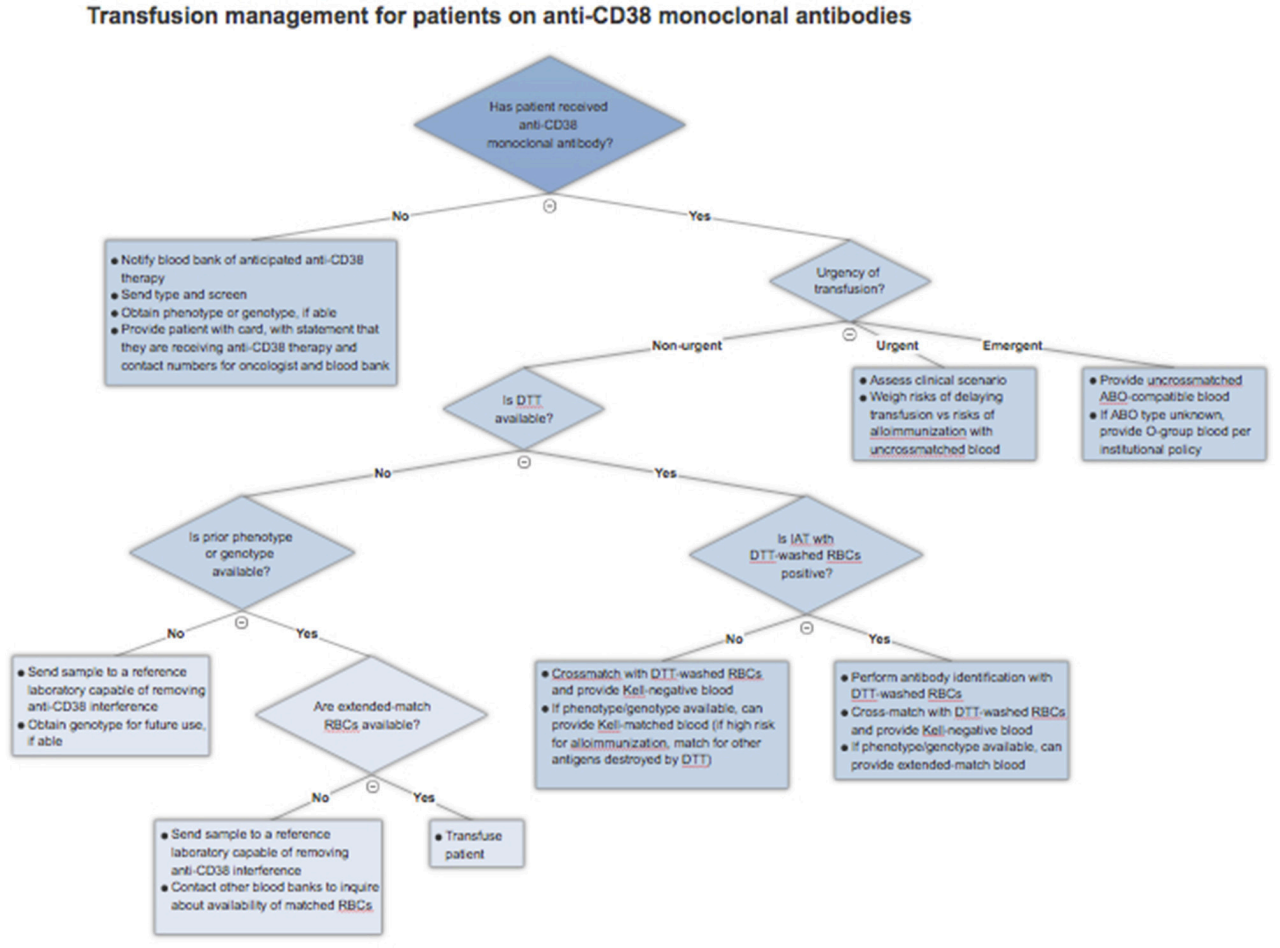
Blood transfusion management algorithm for patients treated with anti-CD38 monoclonal antibodies.

As of yet, there is no universal solution to the problem of anti-CD38 antibody interference in pre-transfusion and RBC compatibility testing. As a result, clinicians must understand the various techniques described and factor in local practices, cost, and availability when approaching this issue. This section will serve as a general guide to approaching blood transfusions in patients receiving anti-CD38 monoclonal antibodies. An algorithm for clinical management is presented in Figure 2.

### Prior to initiation of therapy

Prior to the first dose of an anti-CD38 monoclonal antibody, all patients should get a type and screen to identify any alloantibodies present at baseline. They should also get either an extended phenotype or genotype, depending on the resources available. Incorporating a baseline type and screen and phenotype/genotype into the electronic or written chemotherapy order set can ensure that a type and screen sample is collected and sent to the blood bank along with notification of impending anti-CD38 therapy. Importantly, phenotyping may be inaccurate if a patient has received a blood transfusion in the prior 3 months or if they have a positive DAT; genotyping is unaffected.

This baseline testing ensures that the patient can receive appropriately matched blood even if their local blood bank does not perform DTT or trypsin testing. This is especially valuable in patients who are anticipated to require many transfusions in the future. Ideally, the blood bank would provide the patients with a card detailing their blood type and RBC antigens. However, at a minimum, patients should always carry a card indicating they are receiving anti-CD38 therapy that could interfere with blood typing along with contact information for the treating oncologist and blood bank. This allows other professionals to readily obtain this information in the event of travel or an emergency requiring care away from a cancer center.

### Patients on therapy who require a blood transfusion

If a patient has already received therapy and requires a blood transfusion, the blood bank should be explicitly notified that the patient is on a CD38 monoclonal antibody and requires special testing. Given near universal IAT positivity in this scenario, the blood bank can proceed with DTT treatment of RBCs, if available, as this is currently the most validated method. If there is a negative RBC antibody screen after application of DTT, the patient can be assumed to have no alloantibodies, bearing in mind those antigens, particularly Kell, denatured by the reagent. The blood bank can then crossmatch DTT-treated Kell-negative RBCs with the patient's serum and safely issue compatible blood. There remains a very slim possibility of a transfusion reaction to one of the minor antigens destroyed by DTT treatment, so clinicians should remain alert of this possibility. If the IAT is positive even after application of DTT, this suggests a true alloantibody, and antibody identification should be performed using DTT-treated RBCs.

The availability of an extended RBC phenotype or genotype can simplify this process. Matched RBCs can be issued by the blood bank depending on the extent of matching required and the availability of those units. Not all blood banks will have sufficient inventory to provide extended matches and may wish to preserve these scarce resources for patients known to have multiple alloantibodies. In this case, the blood bank can proceed with DTT-treated RBC antibody screen, with the advantage of knowing ahead of time the patient's Kell status. The blood bank can then provide crossmatched and Kell-compatible blood without the need for extensive antigen matching.

In the case that the blood bank is unable to perform testing with DTT and there is no phenotype or genotype available, a sample should be sent to a reference laboratory that has methods for removing anti-CD38 interference. This will increase the time required to procure appropriate blood, but it is a necessary step to ensure patient safety, provided that there is no urgency for transfusion.

### Emergent and urgent transfusions

Patients who require blood transfusion in a life-threatening situation should receive uncrossmatched ABO and Rh-compatible blood. If the patient's blood type is unknown, they should be given group O red cells, as with other patients. There should not be any delay due to IAT positivity.

In the case of urgent, but not life-threatening, need for transfusions, the clinician must weigh the risks and benefits of providing uncrossmatched blood. In general, the acute risk to the patient is quite low as the likelihood of prior alloimmunization is low. In a large study of emergency-release transfusions involving 1,407 patients and 4,144 units of RBCs, 3% of patients developed alloantibodies after the acute event, while only 0.3% had received incompatible blood and 0.02% had a delayed hemolytic transfusion reaction, with no acute hemolytic reactions reported (32). These rates are likely to be higher in MM patients who have received transfusions previously. The turnaround time required to provide phenotypically-matched RBCs (if patient has a known phenotype or genotype) or performing DTT testing must be considered in the context of the urgency of the clinical situation and the risks of alloimmunization.

## Conclusion

Anti-CD38 monoclonal antibodies are highly efficacious therapies for MM, and their use is likely to increase as they continue to gain new indications. Daratumumab has now been approved in the frontline setting for transplant-ineligible MM and is in late-stage testing for many other malignancies, while isatuximab is in phase three trials for MM. As use of these antibodies becomes routine and expands into the community setting, the challenges and costs associated with blood compatibility testing will continue to grow. It will become imperative for laboratories and medical centers to streamline processes and maintain open communication. To date, there have been no safety issues for these patients receiving transfusions, and it must remain a priority to ensure that patient safety is preserved. This will require the awareness and education of patients, clinicians, and blood bank personnel.

## Author contributions

GL designed and drafted the manuscript; SA, JJ, HC, SJ, DM, SP, and JR critically revised the manuscript; AC conceived, designed, and revised the manuscript. All authors agreed on the final version to be submitted.

### Conflict of interest statement

HC: Research funding: BMS, Janssen, Agenus Inc. Consulting: BMS, Roche. SJ: Advisory Board: Celgene, BMS, Merck, Janssen, Novartis. JR: Advisory Board and Speakers Bureau: Janssen AC: Advisory Board: Adaptive Biotechnology, Amgen, Celgene, Janssen, Millennium/Takeda, Multiple Myeloma Research Foundation, Novartis, Seattle Genetics. Research Funding: Amgen, Array Biopharma, Celgene, Janssen, Millennium/Takeda, Novartis, Pharmacyclics. Consulting: Amgen, Bioascend, BMS, Celgene, Imedex, Janssen, Karyopharm, Millennium/Takeda, Novartis, Physicians Education Resource, The Binding Site. The remaining authors declare that the research was conducted in the absence of any commercial or financial relationships that could be construed as a potential conflict of interest.
